# From semi-conscious to strategic paragraphing

**DOI:** 10.1007/s40037-019-0507-4

**Published:** 2019-04-10

**Authors:** Lorelei Lingard

**Affiliations:** Health Sciences Addition, Schulich School of Medicine & Dentistry, Western University, London, ON Canada

In the Writer’s Craft section we offer simple tips to improve your writing in one of three areas: Energy, Clarity and Persuasiveness. Each entry focuses on a key writing feature or strategy, illustrates how it commonly goes wrong, teaches the grammatical underpinnings necessary to understand it and offers suggestions to wield it effectively. We encourage readers to share comments on or suggestions for this section on Twitter, using the hashtag: #how’syourwriting?

*All writing is a march of paragraphs,*

*each of which provides a clear step forward in the progress of the piece*.

*~Charles Euchner* [1]

Deep in the writer’s subcortex lives the primitive instinct to paragraph. We tap away at our laptop keyboards, periodically hitting the ↵ key to send the cursor back to the left margin. Often this act is only semi-conscious: when our writing session ends, we are as surprised to find our work paragraphed as we are to find our coffee mug empty. Other times, we paragraph visually, hitting ↵ because the paragraph looks too long, or because we want to change topics and we know that white space is the way to signal this on the page. Such semi-conscious paragraphing isn’t necessarily *wrong*, but it is problematic. Paragraphs are not just a structural feature of writing, they are a rhetorical device, arguably the most powerful tool we have for organizing and developing an argument. Not paying them careful attention, therefore, limits the persuasiveness and clarity of our writing. This Writer’s Craft offers a language for thinking strategically about paragraphing and addressing common pitfalls.

## What is a paragraph?

A paragraph is a single unit of thought made up of a group of related sentences. Two principles govern effective paragraphing: unity and coherence. Unity refers to the paragraph’s single main idea, which should be readily identifiable, introduced up front, developed convincingly, and concluded. Coherence refers to the relationships among the sentences in the paragraph. Each sentence should participate in the main idea and be arranged to create the sense of a developing logic rather than a random list.

**Fig. 1:**
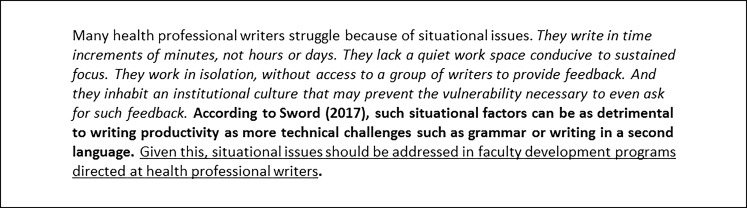
Example of Topic/Body/Token/Wrap Model

A popular model for achieving paragraph unity and coherence in academic writing is the Topic/Body/Tokens/Wrap structure [2]. The first sentence of a paragraph is the Topic sentence; it announces the paragraph’s main idea. *Body sentences* develop that core idea. **Token sentences** are interwoven through the Body, providing illustrative examples or supporting evidence. The Wrap sentence pulls the paragraph argument together and also often provides a link forward to the next paragraph. Fig. 1 illustrates this model.

The Topic/Body/Tokens/Wrap model provides a vocabulary for paying careful attention to paragraphs—your own, or those of the writers you’re supporting. Critical questions we can now ask include:

- Does the first sentence clearly signal the Topic of the paragraph? In the example above, the use of a simple sentence structure [3] (subject, verb, object) helps to ensure that the reader can’t miss the main idea. In paragraphs that develop sophisticated ideas, simple sentences for the Topic and the Wrap can improve clarity.
- Do the Body sentences all develop the main idea, or are some a distraction? Is there a logical pattern to their organization? Pattern is a rhetorical strategy that makes a paragraph more convincing. In the example above, the organizing pattern is one of broadening scope, from the writer’s time and space to their peer group and their institution. Parallel structure [4] is also used to cluster the body sentences elaborating situational issues: ‘They write … They lack … They work … And they inhabit …’.
- Are the Token sentences placed strategically? Not all paragraphs have Token sentences, because not all Body sentences require a Token. In the example above, for instance, there are four Body sentences followed by one supporting Token sentence. There is no rule about the number or positioning of Tokens; however, Dunleavy warns that they are ‘inherently digressive’ and therefore require careful signposting to maintain coherence, particularly when a series of Tokens is used [2]. They may also be a good site for editing when you wish to tighten the argument: ask yourself if all your Tokens are necessary, and delete those that are superfluous or tangential.
- Is there a Wrap sentence? Many paragraphs simply end, without concluding. A good Wrap sentence should not just repeat the Topic sentence, or the paragraph risks feeling that it has not developed meaningfully. In the example above, the Wrap introduces the implications of situational writing issues for faculty development, thus pointing forward to the next paragraph which will elaborate relevant faculty development approaches. Wrap sentences can also be written to elaborate a Topic that was introduced simply. This can help to ensure that, in hindsight, the reader understands the paragraph as *developing* rather than meandering.

## Paragraphing pitfalls

Paragraphing is a challenging skill to develop. Let’s consider some of the ways writers can get off track, and how these problems can be addressed.

### The infinity

paragraph seems to go on forever, straining the reader’s cognitive resources. This strain signals two problems. The first is a problem of internal coherence: i. e., the transitions between sentences within a paragraph that show relationships among them and create a sense of unity. The second is a problem of external coherence: i. e., the connections between this paragraph and the paragraphs that came before it in the paper’s unfolding argument. Readers not only get lost inside the infinity paragraph; they forget how they got to it in the first place. There is no rule to follow to know if your paragraph has gotten too long: the length of a paragraph is determined by the demands of content, not by the space on the page. But generally, if your paragraph is more than a manuscript page in length, ask yourself if it can be broken into two or three paragraphs. Crafted properly, with strong transitions between them to support external coherence, three shorter paragraphs may help you to develop your argument in a more persuasive manner.

### The hiccup

paragraph ends as abruptly as it started, leaving the reader uncertain how—or if—the argument has developed. As British grammarian H.W. Fowler said, ‘The paragraph is essentially a unit of thought, not of length. … A succession of very short ones is as irritating as very long ones are wearisome’ [5]. A very short paragraph can be used to provide emphasis in an argument, but a series of them could be a sign that your ideas are not well-developed. You can think of your paragraphs as links in a chain: the strength of each link is dependent on how well that paragraph develops its idea. If you tend toward hiccup paragraphs, one way to improve is to see if a sequence of them can be combined. Combining them will force you to articulate the relationships among these ideas, which is part of the work of *developing *an idea rather than simply *stating* it.

### The blindfold

paragraph leaves the reader to wander blindly in search of the paragraph’s topic. In what Dunleavy calls the ‘throat clearing intro’ [2], academic writers can begin their paragraphs too broadly as they reference related ideas within the field, making the reader wait until the third or fourth sentence for the main topic. By then, paragraph unity is already threatened, particularly if the reader decided for themselves that something in those first sentences was the topic of interest. To improve a blindfold paragraph, ask yourself if your first few sentences can just be deleted, bringing an existing statement of the main idea into the Topic sentence slot.

### The maze

paragraph has a clear entrance and exit but readers get lost inside it. Sometimes they become so lost that they give up trying to find the logical thread and simply jump to the next paragraph. To improve a maze paragraph, look to the content, number and organization of the Body and Token sentences. Are they all relevant? Is there a logic to their arrangement? Have you signalled the relationships between them through the use of conjunctions (such as ‘but’, ‘or’, ‘and’), adverbs (such as ‘however’, ‘similarly’), and prepositional phrases (such as ‘by contrast’, ‘in comparison’, ‘on the other hand’). These little ‘connecting’ words are essential if your paragraphs are going to ‘climb the arc’ [1], Euchner’s metaphor for strong paragraph development and internal coherence.

### The cliff-hanger

paragraph leaves the reader in mid-air and jumps to the next idea in the argument. Cliff-hangers lack a clear Wrap sentence. In a common example, qualitative research writers often end a paragraph with a quotation. While the right quotation may serve as a Wrap, more often they act as a Token to evidence a specific Body claim. If you want to end with a quotation, ask yourself which function it is serving. Without a Wrap, the writer forfeits two opportunities: both to reinforce what the reader has learned in the paragraph and to signal what is coming next in the argument. Wrap sentences can be used for summary alone or they can also accomplish forward signalling of the next idea in the logical chain; you should consider when each is most effective. Particularly when the next paragraph might otherwise feel like a strange departure from the paper’s logical thread, a forward signalling Wrap can smooth this transition.

## Conclusion

Teased from the subcortex into the light, paragraphing is revealed to be not only structure, but also strategy. This Writer’s Craft has offered a model and some general rules for paragraphing, but there is no single right way to paragraph. Models and rules are very helpful to novice writers, but, once learned, they should be treated as a basis for expert improvisation. So don’t feel tied down. Try using an abrupt, very short paragraph to highlight a critical moment of transition in your argument. Try dispensing with the declarative Topic sentence in favour of a provocative opening question. Try listing multiple Tokens to give the reader the impression of a wealth of evidence for a particular point. The key to success is to uphold the principles of unity and coherence while you play with strategic paragraphing.
